# Diagnostic outcome of ureteroscopy in urothelial carcinoma of the upper urinary tract: Incidence of later cancer detection and its risk factors after the first examination

**DOI:** 10.1186/s12894-015-0086-6

**Published:** 2015-09-04

**Authors:** Norihiro Murahashi, Takashige Abe, Nobuo Shinohara, Sachiyo Murai, Toru Harabayashi, Ataru Sazawa, Satoru Maruyama, Kunihiko Tsuchiya, Naoto Miyajima, Kanako Hatanaka, Katsuya Nonomura

**Affiliations:** Department of Urology, Hokkaido University Graduate School of Medicine, North-15, West-7, North Ward, Sapporo, 060-8638 Japan; Department of Urology, Hokkaido Cancer Center, Sapporo, Japan; Department of Urology, Obihiro-Kosei General Hospital, Obihiro, Japan; Department of Surgical Pathology, Hokkaido University Hospital, Sapporo, Japan

## Abstract

**Background:**

To determine the incidence of later cancer detection and its risk factors after the first diagnostic ureteroscopy.

**Methods:**

One hundred and sixty-six patients undergoing diagnostic ureteroscopy based on the suspicion of urothelial carcinoma of the upper urinary tract (UC of the UUT) between 1995 and 2012 were included. We examined the diagnostic outcome of the initial ureteroscopy. Thereafter, we collected follow-up data on patients who had not been diagnosed with UC of the UUT at the first examination, and evaluated the incidence of later cancer detection and its risk factors using Cox hazard models.

**Results:**

Of the 166 patients, 76 (45.8 %) were diagnosed with UC of the UUT at the first diagnostic ureteroscopy. The remaining 90 (54.2 %) were diagnosed with other malignancies (n = 22), non-malignant disorders (n = 18), or without disorders (n = 50). Of these 90 patients, follow-up data were available in 65 patients (median: 41 months, range: 3–170). During the follow-up, carcinoma was detected in 6 patients (6/65, 9.2 %) at a median of 43.5 months (range: 10–59). Episodes of gross hematuria (p = 0.0048) and abnormal cytological findings (p = 0.0335) during the follow-up and a male sex (p = 0.0316) were adverse risk factors.

**Conclusion:**

Later cancer detection of UC of the UUT was not uncommon after the first examination. The risk analysis revealed the aforementioned characteristics.

## Background

Based on recent advances in medical equipment, ureteroscopy has become a powerful tool for the diagnosis and endoscopic treatment of patients with urothelial carcinoma (UC) of the upper urinary tract (UUT) [1–6]. The combination of direct visual examination and tumor biopsy by endoscopic cold forceps has led to marked diagnostic accuracy. However, there are potential limitations, such as the endoscopic view can be easily compromised by bleeding, and tissue samples obtained using endoscopic forceps are too small to yield a definitive diagnosis regarding the presence or absence of malignancy. In that situation, subsequent follow-up would be necessary. Data regarding these issues have not been reported. In the present study, we evaluated diagnostic outcomes of ureteroscopy and collected follow-up data on patients who were not considered to have UC of the UUT at the first examination. The aim of this study was to clarify the incidence of later cancer detection and its risk factors after the first examination.

## Methods

After obtaining the approval of Institutional Review Board of Hokkaido University Hospital for Clinical Research to access patient data, the medical records of patients undergoing ureteroscopy under general or lumbar anesthesia at Hokkaido University Hospital between 1995 and 2012 were reviewed. During this period, 208 patients underwent ureteroscopic procedures. For the present analyses, patients undergoing ureteroscopy mainly for endoscopic treatment for UC of the UUT, urolithiasis, or other diseases were excluded (n = 16). In addition, because of the special circumstances, patients undergoing ureteroscopy through an antegrade approach, an ileal conduit, or ureterocutaneostomy were excluded (n = 16). Patients under 18 years old (n = 2), those undergoing ureteroscopy for the removal of a migrated stent (n = 3), those with failure on ureteroscopy (n = 4), and a patient undergoing ureteroscopy for suspicion of recurrence after conservative treatment of UC of the UUT at the previous hospital were also excluded. Finally, 166 patients undergoing diagnostic ureteroscopy to obtain a diagnosis of UC of the UUT were included. Regarding the indication of diagnostic ureteroscopy, patients with abnormal radiological findings, such as hydronephrosis, a solid mass within the urinary tract, gross hematuria originating from the upper urinary tract, or positive urine cytology with a normal bladder mucosal appearance were considered to be candidates. In patients with apparent imaging findings and positive urinary cytology, we generally proceeded with radical surgery without diagnostic ureteroscopy.

### Details of procedure

Before ureteroscopy, almost all patients underwent cystoscopy, CT, and voided urine cytology at our outpatient clinic. Under general (n = 86) or lumbar (n = 80) anesthesia, we initially performed cystoscopy and, thereafter, observed the upper urinary tract using a semi-rigid ureteroscope. Since 1998, flexible ureteroscopy has also been available in our hospital. Although, during the study period, several models of ureteroscopes were used due to the introduction of new models or simply the wear and tear of equipment, a semi-rigid ureteroscope of Richard Wolf (size: 6.0-7.5 Fr, working channel: 4 Fr) and a flexible ureteroscope of Olympus (size: 5.3-8.4, working channel: 3.6 Fr) were mostly used. With the use of 3 Fr forceps, biopsy of any suspicious region was performed, and samples were processed in formalin fixative. Washing urine samples were also collected. In patients with abnormal cytological findings without apparent abnormal radiological findings, random biopsy of the bladder mucosa was also conducted.

In the present study, we examined the diagnostic outcome at the initial ureteroscopy. Thereafter, we collected follow-up data on patients who had been diagnosed without UC of the UUT, and evaluated the incidence of later cancer detection and associated risk factors.

### Statistical analysis

Cox proportional hazard model addressed the association between the clinical characteristics and later cancer detection. Survival probabilities were estimated using Kaplan-Meier methods, and survival distributions were compared with the log-rank test. All calculations were performed using JMP version 11. P-values < 0.05 were considered significant.

## Results

Table 1 shows the patients’ characteristics. The median age was 67.5 years (range: 22–89). Of the 166 patients, 118 (71.1 %) underwent diagnostic ureteroscopy based on abnormal radiological findings, 76 (45.8 %) based on abnormal cytology findings, and 78 (47.0 %) due to macrohematuria (there were overlaps among the groups). In the present cohort, 55 (33.1 %) patients had a concurrent or previous history of bladder cancer.

**Table 1:** Patients’ characteristics

	n = 166
Age, years	Median: 67.5 (range: 22–89)
Sex male/female	
Male	107 (64.5 %)
Female	59 (35.5 %)
Side evaluated by ureteroscopy	
Unilateral	143 (86.1 %)
Bilateral	23 (13.9 %)
Reason for undergoing ureteroscopy	
Abnormal radiological finding only	39 (23.5 %)
Abnormal cytological finding only^a^	21 (12.7 %)
Macrohematuria only	13 (7.8 %)
Multiple reasons any of the above 3 indications	83 (50 %)
Missing information	10(6 %)
Concurrent or previous history of bladder cancer	
Yes	55 (33.1 %)
No	111 (66.9 %)

^a^Abnormal cytological finding means malignant, suspicious, or atypical cells

**Fig. 1:**
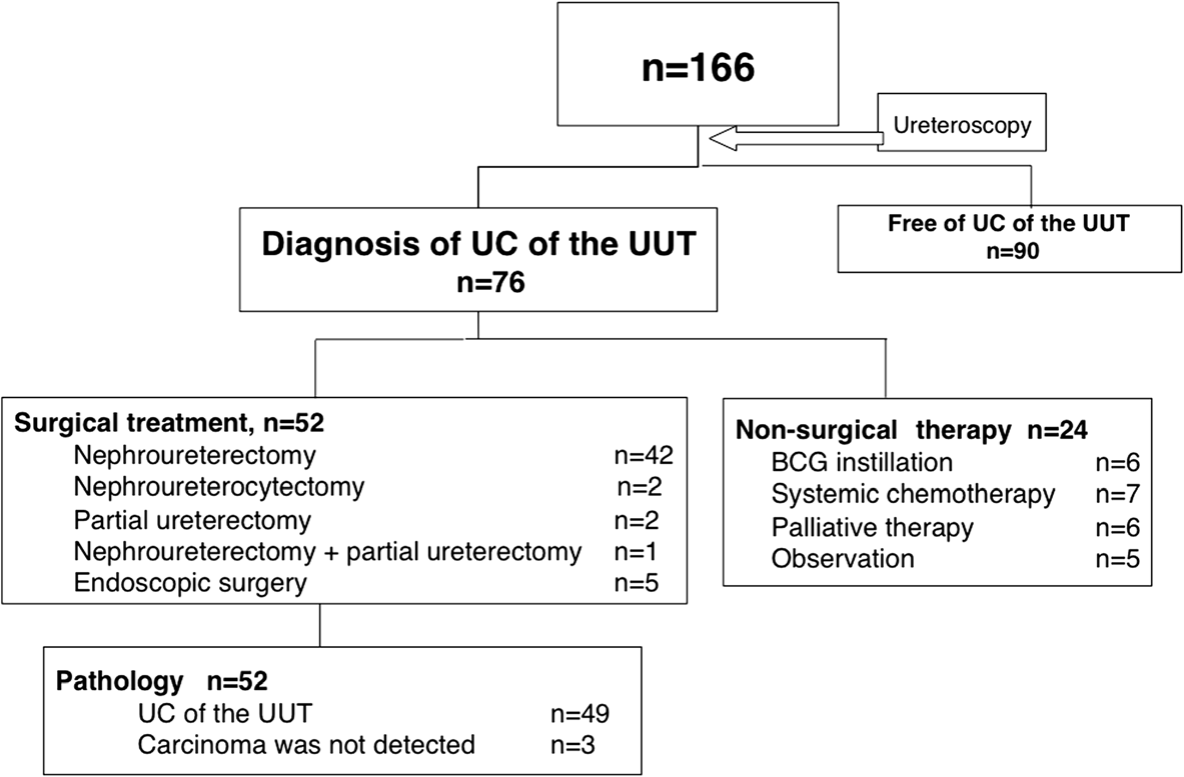
Summary of the first examination. UC = urothelial carcinoma. UUT = upper urinary tract

**Fig. 2:**
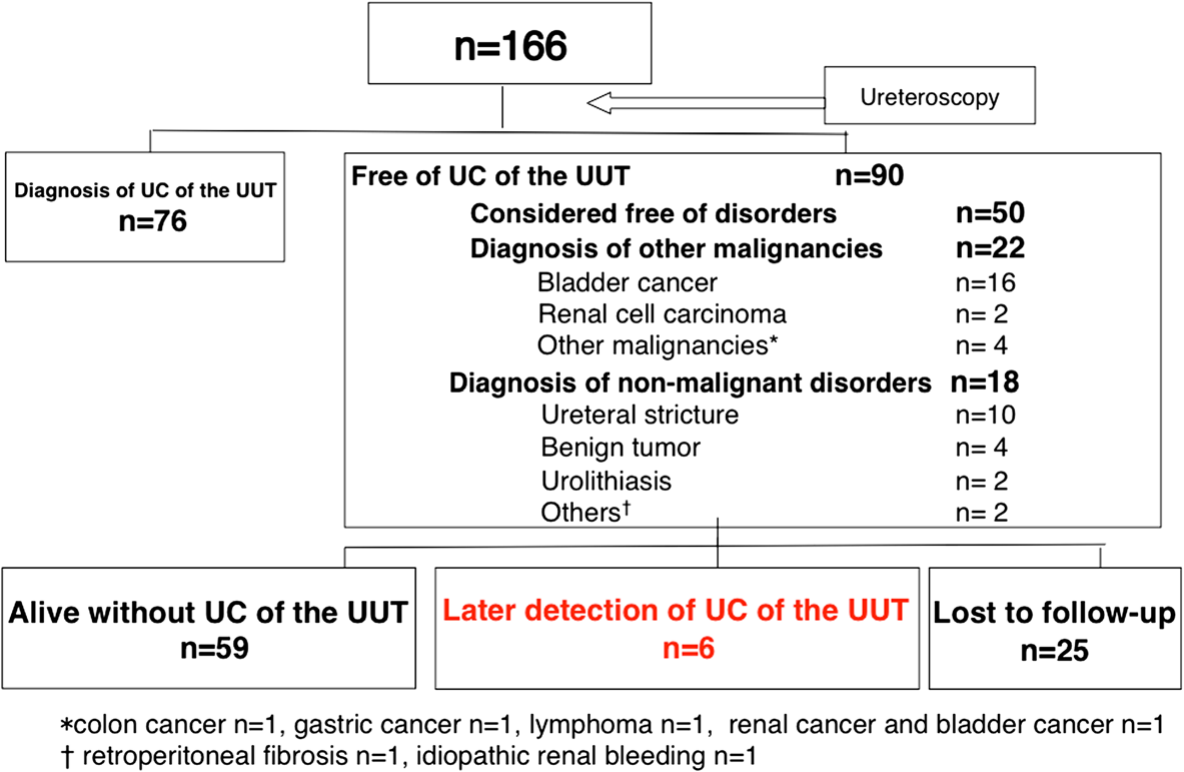
Summary of the remaining 90 patients without UC of the UUT at the first examination

Figure 1 summarizes the diagnostic outcomes of initial examinations. Of the 166 patients, UC of the UUT was detected in 76 (45.8 %) patients. After the diagnosis, 42 patients underwent nephroureterectomy, 2 underwent nephroureterocystectomy, 2 underwent partial ureterectomy, 1 patient with bilateral UC of the UUT underwent nephrouretectomy and contralateral partial ureterectomy, and 5 underwent endoscopic conservative surgery. Pathological examination after surgery revealed that 49 patients had UC of the UUT, while 3 patients did not show evidence of carcinoma in the surgical specimens. The remaining 24 patients underwent non-surgical treatment (BCG instillation into upper urinary tract: n = 6, systemic chemotherapy: n = 7, palliative therapy: n = 6, and observation: n = 5). Figure 2 summarizes the diagnostic outcomes of the remaining 90 patients without UC of the UUT at the first examination. Fifty patients, in whom no apparent tumor was observed on ureteroscopic evaluation or pathological evaluation, and washing cytology did not lead to a definitive diagnosis of UC, were considered to be without malignancy or urological disorder. In 22 patients, malignant diseases other than UC of the UUT (bladder cancer: n = 16, renal cell carcinoma: n = 2, other malignancies n = 4) were detected. In addition, non-malignant disorder was detected in 18 patients (ureteral stricture: n = 10, benign tumor: n = 4, urolithiasis: n = 2, others: n = 2). Regarding the complications among the 166 patients, major ureteral injury occurred in one patient with a ureteral stone and severe hydronephrosis, which later resulted in nephrectomy. Minor ureteral injury occurred in 7 patients, which was resolved by ureteral stent placement. No urosepsis occurred after ureteroscopy.

After the first ureteroscopy, follow-up data were available in 65 patients with a median 41-month (range: 3–170 months) follow-up duration, while 25 patients were lost to follow-up. During the follow-up period, 11 patients underwent a second ureteroscopy, and UC of the UUT was detected in 5 patients. An additional patient developed metastatic urothelial carcinoma 33 months after the first examination (case No. 6 in Table 2). Therefore, UC of the UUT was detected in a total of 6 patients (6/65, 9.2 %) at a median of 43.5 months (range: 10–59 months) after the first ureterosopy (Fig. 2 and Table 2). Table 3 summarizes the results of uni- and multivariate analyses of risk factors for later cancer detection. Episodes of gross hematuria (p = 0.0048) and abnormal cytological findings (p = 0.0335) during the follow-up and a male sex (p = 0.0316) were adverse risk factors of later cancer detection. When using a multivariate model adjusting for episodes of gross hematuria and abnormal cytological findings, episodes of gross hematuria remained significant (hazard ratio: 7.84, 95 % confidence interval: 1.32-61.7, p = 0.0239).

**Table 2:** Summary of the 6 patients with later detection of UC of the UUT

Case No.	Age, years	Sex	Side of first examination	Mucosa appearance of upper urinary tract at first examination	Washing cytology at first examination	Ureteral biosy at first examination	Diagnosis at first examination
1	55	Male	L	normal	not performed	not performed	bladder cancer
2	71	Male	R	normal	negative	negative	free of disorders
3	82	Male	R	normal	atypical	not performed	bladder cancer
4	68	Male	R	irregular	negative	negative	bladder cancer
5	70	Male	L	normal	negative	not performed	free of disorders
6	85	Male	L	normal	atypical	negative	free of disorders
Case No.	Side of subsequent cancer	Episode of gross hematuria after first examination	Urine cytology after first examination	Diagnostic method	Interval between first examination and cancer detetion	Treatment	Pathology
1	L	No	negative	ureteroscopy	57	nephroureterectomy	UC,G3 > 2,pT3
2	R	Yes	negative	ureteroscopy	10	nephroureterectomy	UC,G1 > 2,pT2
3	R	No	negative	ureteroscopy	60	nephroureterectomy	UC,G3,pT3
4	R	Yes	suspicious	ureteroscopy	28	BCG	UC, G2 > G3, pTa
5	B	Yes	positive	ureteroscopy	55	BCG for CIS of UUT	-
6	L	Yes	suspicious	CT	33	palliative therapy	-

**Table 3:** Univariate and multivariate analysis of risk factors for later cancer detection

Factor	No. of patients	Univariate hazard ratio (95 % confidence interval)	P-value	Multivariate hazard ratio (95 % confidence interval)	P-value
Age, year					
continuous	65	1.07 (0.99-1.18)	0.0807		
Gross hematuria after first examination					
Yes	13	11.3 (2.15-82.8)	0.0048	7.84 (1.32-61.7)	0.0239
No	50	1		1	
Cytology after first examination					
Positive/suspicious/atypical	9	6.6 (1.18-37.0)	0.0335	4.58 (0.689-31.3)	0.112
Negative	47	1		1	
Concurrent or previous history of bladder cancer					
Yes	22	3.09 (0.6-22.4)			
No	43	1	0.177		
Smoking history					
Yes	32	4.08 (0.66-78.1)			
No	26	1	0.142		
Sex		5-year cancer-free survival rate, %			
Male	40	73			
Female	25	100	0.0316		

## Discussion

In the present study, 76 (45.8 %) of the 166 patients were diagnosed with UC of the UUT at the first examination. Although the detection rate of UC of the UUT was lower than in previous studies [5, 6], we consider that it is strongly influenced by the indication of diagnostic ureteroscopy at each institution. As aforementioned, we proceed directly to radical surgery without ureteroscopy in patients showing apparent imaging findings with a positive urinary cytology, and this would contribute to our lower detection rate. The incidence of urinary stones was low in our cohort, because patients requiring stone treatment were usually referred to our teaching hospitals.

At the initial diagnosis, UC of the UUT was not detected in surgical specimens in 3 patients (5.8 %, 3/52). The final pathology revealed dysplasia in one patient and the remaining two patients had neither carcinoma nor dysplasia. Of these three patients, one was diagnosed with a pelvic tumor due to positive washing cytology. This patient had concurrent bladder carcinoma, and contamination by carcinoma cells from bladder cancer would lead to a misdiagnosis. The remaining two patients were diagnosed by mucosal biopsy, which would suggest the difficulty of pathological diagnosis using small biopsy samples. Tsivian et al. reported a similar rate of misdiagnosis (not UC based on final pathologic findings), whereby it was 2.1 % (1/48) with routine ureteroscopic assessment [5]. Interestingly, they reported that the rate of misdiagnosis was 15.5 % (9/58) before routine ureteroscopic evaluation, which suggested improvement of the diagnostic accuracy due to ureteroscopy.

After the first ureteroscopy, follow-up data were available in 65 patients with a median of 41 months (range: 3–170 months), and UC of the UUT was detected on second ureteroscopy in 5 patients. Because one additional patient developed metastatic urothelial carcinoma detected by CT, UC of the UUT was detected in a total of 6 patients (6/65, 9.2 %) at a median of 43.5 months (range: 10–59 months) after the first ureteroscopy, which was an unexpectedly high detection rate. Regarding Case 2 in Table 2, because the interval between the first ureteroscopy and definitive diagnosis was relatively short (10 months), we considered that UC of the UUT carcinoma might be missed at the first examination. In the remaining 5 patients, because UC of the UUT was diagnosed after more than two years (range: 28–60 months), these carcinomas might be de novo development rather than being missed at the first examination. Cases 1, 3, and 4 had concurrent bladder cancer, and it is well-known that patients with bladder cancer are at risk of upper urinary tract recurrence. Picozzi et al. reported in their meta-analysis that the incidence of upper urinary tract recurrence after cystectomy ranged from 0.75 to 6.4 % [7]. However, interestingly, the laterality of the carcinoma was the same as that observed at the first examination in all 6 cases, although we could not clarify the precise mechanism. At present, we consider our observations to suggest that later cancer detection of UC of the UUT was not uncommon after the first examination, but this should be verified in another cohort.

Regarding the risk factors of later cancer detection, the univariate model identified episodes of gross hematuria (p = 0.0048) and abnormal cytological findings (p = 0.0335) during the follow-up and a male sex (p = 0.0316) as adverse risk factors. Regarding the sex difference, previous epidemiologic studies revealed conflicting observations of a male [8–10] or a female [11] predominance in the incidence of UC of the UUT. Alternatively, a difference in accessibility to the upper urinary tract between males and females, due to differences in the urethral length, may influence the outcome. In the present study, the hazard ratio of males to females could not be calculated due to the absence of later cancer detection in the female cohort. When adjusting for episodes of gross hematuria and abnormal cytological findings in the multivariate model, episodes of gross hematuria remained significant (hazard ratio: 7.84, 95 % confidence interval: 1.32-61.7, p = 0.0239).

This study had several limitations, including its retrospective design, small sample size, and variations in ureteroscopies, as well as each surgeon’s experience and proficiency during the study periods. In addition, we could not follow all patients after the first examination and did not have a uniform follow-up protocol, such as an indication for repeat ureteroscopy. Nevertheless, we consider that several important findings were yielded by the present study.

## Conclusion

Later cancer detection of UC of the UUT was not uncommon after the first examination. Risk analysis revealed that episodes of gross hematuria (p = 0.0048) and abnormal cytological findings (p = 0.0335) during the follow-up and a male sex (p = 0.0316) were adverse risk factors.

## Abbreviations

UC: Urothelial carcinoma
UUT: Upper urinary tract
